# Fluorescence Modulation of Green Fluorescent Protein Using Fluorinated Unnatural Amino Acids

**DOI:** 10.3390/molecules22071194

**Published:** 2017-07-16

**Authors:** Jordan K. Villa, Hong-Anh Tran, Megha Vipani, Stephanie Gianturco, Konark Bhasin, Brent L. Russell, Elizabeth J. Harbron, Douglas D. Young

**Affiliations:** Department of Chemistry, The College of William & Mary, Williamsburg, VA 231871, USA; jkvilla27@gmail.com (J.K.V.); hntran@email.wm.edu (H-.A.T.); mavipani@email.wm.edu (M.V.); slgianturco@email.wm.edu (S.G.); kbhasin@masonlive.gmu.edu (K.B.); brent.russell64@gmail.com (B.L.R.)

**Keywords:** unnatural amino acids, green fluorescent protein, biosensors, fluorotyrosine

## Abstract

The ability to modulate protein function through minimal perturbations to amino acid structure represents an ideal mechanism to engineer optimized proteins. Due to the novel spectroscopic properties of green fluorescent protein, it has found widespread application as a reporter protein throughout the fields of biology and chemistry. Using site-specific amino acid mutagenesis, we have incorporated various fluorotyrosine residues directly into the fluorophore of the protein, altering the fluorescence and shifting the pKa of the phenolic proton associated with the fluorophore. Relative to wild type GFP, the fluorescence spectrum of the protein is altered with each additional fluorine atom, and the mutant GFPs have the potential to be employed as pH sensors due to the altered electronic properties of the fluorine atoms.

## 1. Introduction

**Figure 1:**
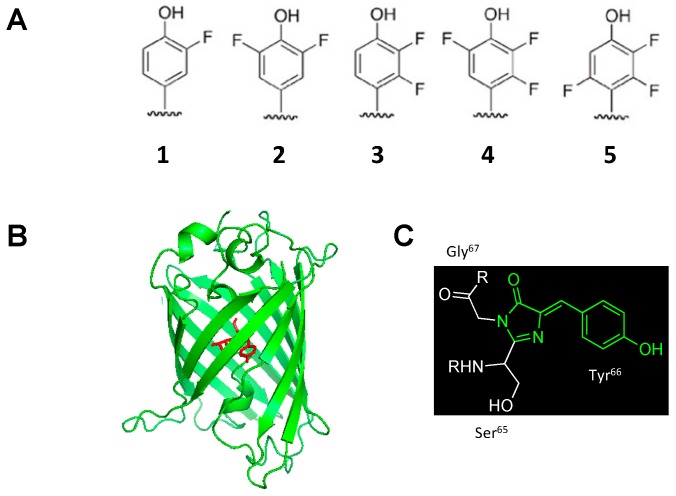
Fluorotyrosines, GFP, and fluorophore structure. (**A**) The structures of the five different fluorotyrosines incorporated into the 66 position of GFP; (**B**) Crystal structure of GFP with the fluorophore highlighted in red at the center of the β-barrel in green [16]; (**C**) Chemical structure of the GFP fluorophore arising from the condensation of the three key amino acid residues.

Fluorescent protein biosensors have become widely utilized to monitor cellular activity in real-time via interactions of analytes with the protein [1,2,3]. In particular, green fluorescent protein (GFP) has become a ubiquitous protein reporter system due to its physiological stability and extensively studied fluorescent properties [4,5,6,7]. Originally isolated from a deep-sea jellyfish, *Aequorea victoria*, the protein’s fluorescence arises from the condensation of a serine, a tyrosine, and a glycine at residues 65–67 to form a chromophore within the center of a β-barrel (Figure 1) [8,9]. Due to the presence of this conserved tyrosine residue at position 66 within the fluorophore, the fluorescence of GFP is modulated by the protonation state of the phenol moiety. Deprotonation leads to increased conjugation and a stronger fluorescent emission at 512 nm, whereas the protonated state emits at approximately 460 nm [8]. This feature suggests that GFP may be useful as a physiological pH sensor, and it has previously been investigated towards this application. 

One mechanism to make GFP more useful as a sensor is tuning the pKa of the phenolic proton. This can readily be accomplished with minor structural perturbation via the substitution of the tyrosine residue with an unnatural amino acid (UAA) [10]. Substitution with a UAA is feasible utilizing the Schultz methodology for site-specific UAA incorporation in vivo, providing mutant GFP proteins directly at residue 66 [11]. This approach has been employed in multiple applications to introduce over 100 structurally different UAAs into a variety of proteins [12,13]. The technology involves the introduction of an orthogonal aminoacyl-tRNA synthetase (aaRS)/tRNA pair that has been evolved to recognize a specific UAA and charge the corresponding tRNA for amino acid incorporation in response to a TAG codon in the mRNA transcript [14,15]. Thus, if a component of the translational machinery is not correctly functioning, the ribosome encounters a stop codon and terminates translation. However, if all components are properly introduced to the expression organism, in the presence of the UAA, functional protein harboring the UAA in a specific site is expressed. 

Fluorotyrosine UAAs are of particular interest in the tuning of GFP fluorescence because they are isosterically similar to the natural tyrosine residue, but have dramatically different electronic properties (Table 1). Previously, 3-fluorotyrosine (**1**) has been employed to determine the structure and kinetics of proteins such as hemoglobin [17], organophosphate hydrolase [18], and human manganese superoxide dismutase [19]. Moreover, its ability to promote and propagate radicals has been explored in the study of numerous reactive tyrosine radical mechanisms [20]. With iterative addition of fluorines into the tyrosine core, the properties of the residue can even further be manipulated by the increase in electronegativity garnered by the fluorine addition. Previous methods to modulate GFP using fluorotyrosines have involved a photocaged fluorotyrosine to introduce difluorotyrosines into GFP using a caged tyrosine aaRS, altering the fluorescent properties of the protein [21]. However, this strategy required UV irradiation of the protein to remove the *o*-nitrobenzyl caging group, which may result in photobleaching of the fluorescent protein, as well as introducing reactive and highly absorbing nitrosoaldehydes into the solution [22]. More recently, a novel aaRS that exhibits a degree of polyspecificity for multiple fluorotyrosine UAAs was evolved and employed to introduce these residues into ribonuclease reductase to study the generation of tyrosyl radicals [10,23,24]. Utilizing this aaRS, it now becomes feasible to incorporate mono-, di-, and trifluorotyrosines into GFP, having an even more dramatic effect on its fluorescent properties. Even more interesting is their effects on the pKa of the phenol, shifting it from the native pKa of 10 to more physiologically relevant pHs. Consequently, we envision exploiting this aaRS to express a series of mutant GFPs and investigate their photophysical and physiological sensing properties for utilization as fluorescent protein biosensors.

## 2. Results and Discussion

**Figure 2:**
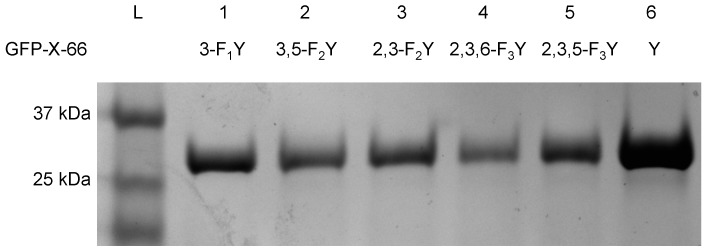
SDS-PAGE analysis of the mutant GFP expressions.

In order to investigate the effect of fluorotyrosine residues on GFP fluorescence, mutant proteins were expressed using *E. coli* cells co-transformed with a pEVOL-3FY plasmid harboring both the aaRS and the tRNA, and a pET-GFP-TAG66 plasmid containing the GFP gene with a TAG codon introduced at residue 66. Due to the ability of the aaRS to recognize and incorporate multiple fluorotyrosine derivatives, these cells could be utilized for the expression of all five mutant proteins. The cells were induced with additions of isopropyl β-d-1-thiogalactopyranoside (IPTG) and arabinose to express GFP in the presence or absence of the fluorotyrosines: 3-fluorotyrosine (3-F_1_Y, **1**), 3,5-difluorotyrosine (3,5-F_2_Y, **2**), 2,3-difluorotyrosine (2,3-F_2_Y, **3**), 2,3,5-trifluorotyrosine (2,3,5-F_3_Y, **4**), and 2,3,6-trifluorotyrosine (2,3,6,-F_3_Y, **5**) (Figure 1). A control expression was also performed with wild-type GFP that contained the standard tyrosine at residue 66. Gratifyingly, full length GFP was obtained only in expression cultures grown in the presence of one of the fluorotyrosine UAAs, whereas no detectable protein was obtained in the absence of the UAA (Supplementary Material, Figure S1). As this is a previously evolved aaRS, the presence of protein and spectral shifts confirm incorporation of the fluorotyrosines. The proteins were then purified and analyzed by gel electrophoresis and mass spectrometry to confirm appropriate molecular weight and purity (Figure 2, Supplementary Material Figure S2).

**Figure 3:**
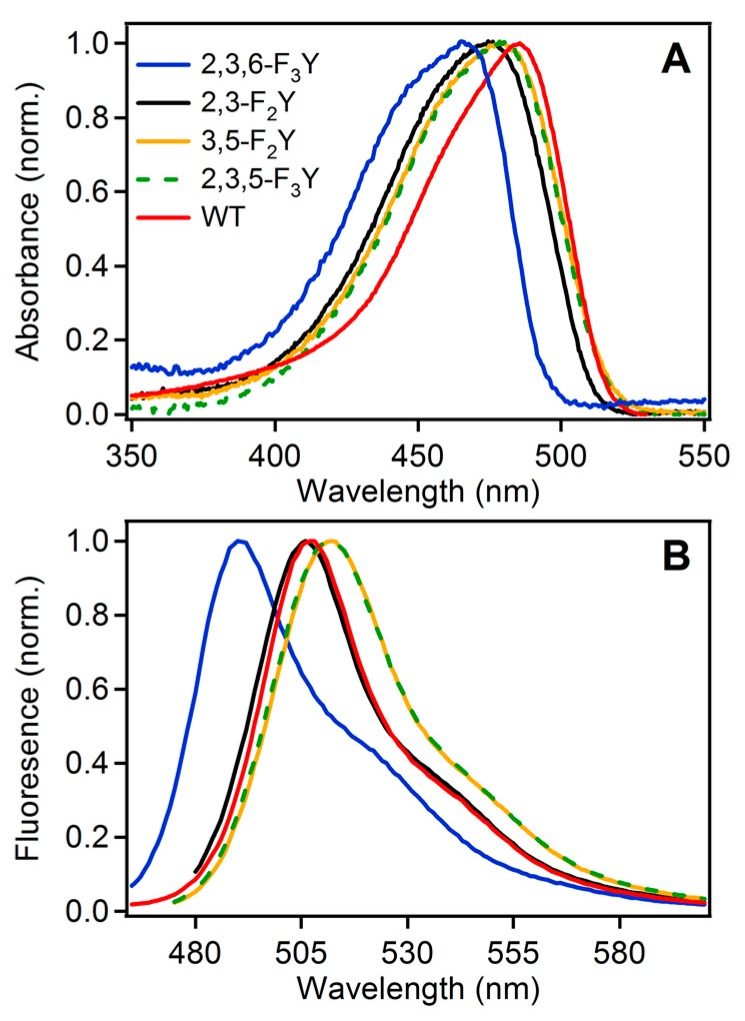
Absorption and fluorescence spectra of the GFP mutants. (**A**) Absorption spectra of the four GFP mutants compared to wild-type; (**B**) Fluorescence spectra of the 4 GFP mutants compared to wild-type.

With the four fluorotyrosine sf-GFP (super-folded green fluorescent protein) mutants in hand, we next investigated their fluorescent properties. It is important to note that this particular GFP has been previously engineered to favor the deprotonated state, thus allowing us to examine the electronic effect of the fluorine substituents directly on the phenolate form without adding confounding spectral data associated with the protonated phenol state. To identify potential spectral shifts in their fluorescence, absorbance measurements were first conducted (Figure 3A). The wild-type GFP exhibited the most red-shifted absorbance with a λ_max_ at 483 nm, in accordance with literature values (Table 2). With the subsequent addition of fluorine substituents to the fluorophore, a blue-shift was observed, with the greatest shift occurring for the 2,3,6-F_3_Y mutant (λ_max_ = 465 nm). The addition of subsequent electron withdrawing fluorines has previously been demonstrated in various aromatic chromophores, and may lead to a stabilization of the HOMO, resulting in an increased energy required for the electronic transitions. After obtaining the λ_max_ values for each mutant, we next sought to investigate their fluorescent properties. Exciting the proteins at the appropriate wavelengths obtained by absorbance measurements, emission spectra were obtained for each GFP mutant (Figure 3B). The most dramatic fluorescent shift relative to the wild-type GFP was again observed with the 2,3,6-F_3_Y mutant, which was significantly blue-shifted. Interestingly, the 2,3,5-F_3_Y and the 3,5-F_2_Y mutants generated a red-shift in the emission spectrum, while the 2,3-F_2_Y was slightly blue-shifted from the wild type protein (Table 2). We hypothesize that the inclusion of two fluorine substituents in the ortho-positions to the phenol may result in a stabilization of the HOMO, as the fluorines are able to better stabilize the negative charge buildup on the ortho-carbons. This negative charge buildup is only stabilized in one resonance form for the 2,3-F_2_Y and 2,3,6-F_2_Y mutants, potentially contributing to the observed blue shift. Again, with this particular GFP protein, we are observing the spectra attributed to the deprotonated tyrosine residue so all shifts can primarily be correlated to the effect of the fluorine substituents and not the protonation state of the phenol.

**Figure 4:**
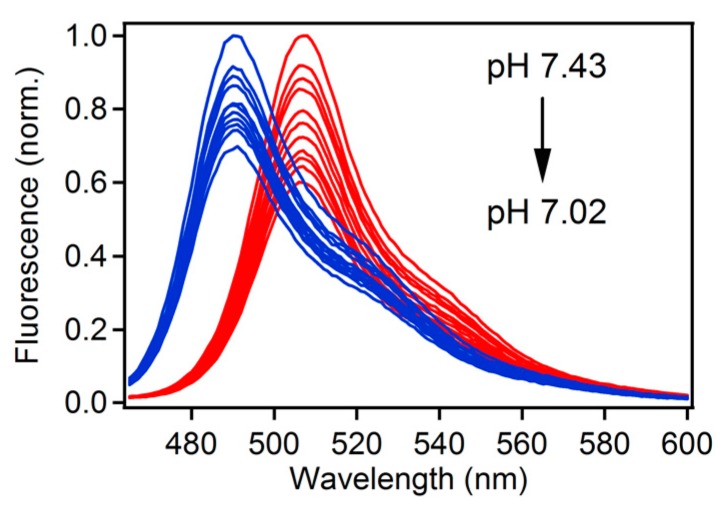
Fluorescence spectra wild-type GFP (red) and 2,3,6-F_3_Y GFP (blue) when titrated with 0.1 M HCl. The pH difference between each measurement was approximately 0.07 pH units.

Due to the potential electronic alterations to the phenolic tyrosine, each mutant may exhibit a unique fluorescent response to pH changes. Motivated by the significant spectral differences, we selected the 2,3,6-F_3_Y mutant to compare to the wild-type protein to see if a fluorinated GFP mutant could potentially act as pH sensors under physiologically relevant conditions. Each protein was titrated with HCl, measuring the new fluorescence spectrum and the pH with each subsequent addition (Figure 4). In order to remain biologically relevant, the pH was only modulated between 7.4 and 7.0, and each addition of 0.1 M HCl attempted to alter the pH by approximately 0.07 pH units. In both cases, the fluorescence intensity decreases as the pH is lowered; however, the change in fluorescence intensity is dependent upon which GFP mutant is employed. The fluorescence of 2,3,6-F_3_Y mutant was more stable to pH changes, losing only 28% of its original intensity in this pH range. However, the WT was much more sensitive to pH change, losing 40% of its initial intensity. While this effect may be specific to this particular GFP mutant (which is optimized to favor the phenolate state), it does indicate that the incorporation of a fluorotyrosine substituent can indeed impact both the fluorescence spectra and the pH sensitivity of the entire protein, demonstrating their potential to be employed as biological sensors. 

## 3. Materials and Methods

### 3.1. General

Solvents and reagents were obtained from either Sigma-Aldrich (St. Louis, MO, USA) or Fisher Scientific (Pittsburgh, PA, USA) and used without further purification. All GFP proteins were purified according to manufacturer’s protocols using a Qiagen Ni-NTA Quik Spin Kit (Qiagen, Boston, MA, USA). 

### 3.2. Expression of Fluorotyrosine Containing GFP Escherichia coli

BL21(DE3) cells were co-transformed with a pET-GFP-TAG-66 plasmid (0.5 μL) and pEVOL-3FY plasmid (0.5 μL) using an Eppendorf electroporator (Eppendorf, Hauppague, NY, USA). Cells were then plated on LB-agar plates supplemented with ampicillin (50 mg/mL) and chloramphenicol (34 mg/mL) and grown at 37 °C. After 16 h, a single colony was selected and used to inoculate LB media (4 mL) supplemented with ampicillin and chloramphenicol. The culture was grown at 37 °C for 12 h. The culture was used to initiate an expression culture of LB media (10 mL) at OD_600_ 0.1, then incubated at 37 °C, to an OD_600_ of ~0.6, at which point cells were induced with 1 M IPTG (10 μL), 20% arabinose (10 μL) and 100 mM (100 μL) of respective fluorotyrosine (3-FY; 2,3-F_2_Y; 3,5-F_2_Y; 2,3,5-F_3_Y; 2,3,6-F_3_Y). Cultures were grown for an additional 16 h at 37 °C, then harvested by centrifugation (10 min at 10,000 rpm). The media was removed and the cell pellet placed in the −80 °C freezer for at 20 min. Purification was accomplished using commercially available Ni-NTA spin columns and according to manufacturer’s protocol. Protein yield and purity was assessed by SDS-PAGE, and with a Nanodrop spectrophotometer (ThermoFisher, Pittsburgh, PA, USA).

### 3.3. Fluorescence Measurements

Fluorescence was measured with a Varian Cary Eclipse Fluorescence Spectrophotometer (Agilent, Santa Clara, CA, USA). Samples were prepared by a 1:100 dilution with PBS buffer or 1× Tris Buffer with aliquots of 0.1 M HCl. At each increment, the pH was measured using a Mettler Toledo microelectrode pH probe (Mettler Toledo, Columbus, OH, USA). Data analysis to test for titration curves was done using IGOR software program (Version 6.05, Wavemetrics, Lake Oswego, OR, USA).

## 4. Conclusions

Overall, we have exploited unnatural amino acid technologies to elucidate the unique spectral properties of both 2,3,5-F_3_Y and 2,3,6-F_3_Y GFP-66 mutants that have not been previously reported. Interestingly, a dramatic hypsochromic shift was observed in both the absorbance and fluorescence spectra of the 2,3,6-F_3_Y GFP-66 relative to wild-type. Moreover, we investigated the pH sensitivity of the fluorescence spectra of the mutants, potentially demonstrating their utility as biological sensors. This work demonstrates the ability to modulate protein function through very mild structural changes as a mechanism to engineer novel function into proteins. Future work will investigate tetra-substituted fluorotyrosine mutants as well as other GFP mutants in their function as in vivo pH biosensors.

## Figures and Tables

**Table 1:** Fluorotyrosine analogs and corresponding pKa values [20].

Tyrosine Derivative	pKa
Y	10
3-F_1_Y	8.4
3,5-F_2_Y	6.8
2,3-F_2_Y	7.6
2,3,5-F_3_Y	6.1
2,3,6-F_3_Y	6.6
2,3,5,6-F_4_Y	5.2

**Table 2:** Spectral properties of fluorotyrosine containing GFP mutants.

GFP	λ_max__,abs_ (nm)	ε (cm^−1^ M^−1^)	λ_max,em_^a^ (nm)
Y	483	61,700	507
3,5-F_2_Y	478	31,400	511
2,3-F_2_Y	473	27,000	506
2,3,5-F_3_Y	478	59,900	512
2,3,6-F_3_Y	465	26,800	491

^a^ Fluorescence excitation wavelengths near the absorbance λ_max_ were used.

## Supplementary Materials

The following are available online, Figure S1: SDS-PAGE analysis of protein expression in the presence and absence of fluortyrosine. Figure S2: LC-MS of fluorotyrosine GFP expressions.
