# Supplementary material for: Fluorescence Modulation of Green Fluorescent Protein Using Fluorinated Unnatural Amino Acids
Source: Molecules. 2017 Jul 16;22(7):1194. doi: 10.3390/molecules22071194 (PMC5806519; doi:10.3390/molecules22071194)
Supplement: Supplementary file 1 [file molecules-22-01194-s001.pdf]

Supplementary Material

Fluorescence Modulation of Green Fluorescent Protein Using Fluorinated Unnatural Amino Acids

Jordan K. Villa, Hong-Anh Tran, Megha Vipani, Stephanie Gianturco, Konark Bhasin, Brent L. Russell, Elizabeth J. Harbron* and Douglas D. Young*

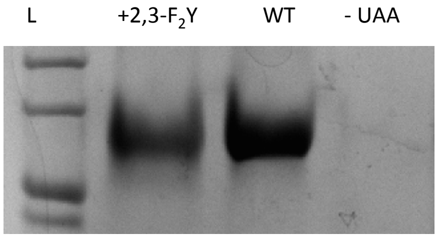

Figure S1. SDS-PAGE analysis of protein expression in the presence and absence of fluorotyrosine. Expressions induced with IPTG and arabinose in the absence of exogenous amino acid resulted in no GFP expression, confirming that the observed proteins harbour fluorotyrosine variants.

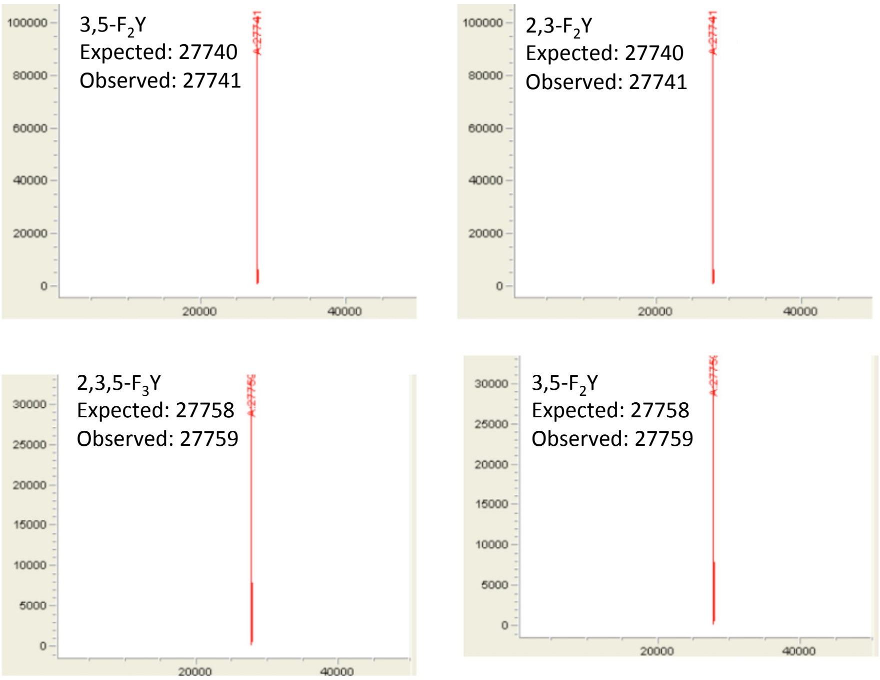

Figure S2. LC-MS of fluorotyrosine GFP expressions.
